# Supplementary material for: Facing flood disaster: A cluster randomized trial assessing communities’ knowledge, skills and preparedness utilizing a health model intervention
Source: PLoS One. 2022 Nov 28;17(11):e0271258. doi: 10.1371/journal.pone.0271258 (PMC9704659; doi:10.1371/journal.pone.0271258)
Supplement: Attachment — Submitted filename: PLOS ONE Review December 16, 2021.pdf [file pone.0271258.s004.pdf]

* Comments to the Author

1. Is the manuscript technically sound, and do the data support the conclusions?

The manuscript must describe a technically sound piece of scientific research with data that supports the conclusions. Experiments must have been conducted rigorously, with appropriate controls, replication, and sample sizes. The conclusions must be drawn appropriately based on the data presented.

YES

*2. Has the statistical analysis been performed appropriately and rigorously?

YES

*3. Have the authors made all data underlying the findings in their manuscript fully available?

The PLOS Data policy requires authors to make all data underlying the findings described in their manuscript fully available without restriction, with rare exception (please refer to the Data Availability Statement in the manuscript PDF file). The data should be provided as part of the manuscript or its supporting information, or deposited to a public repository. For example, in addition to summary statistics, the data points behind means, medians and variance measures should be available. If there are restrictions on publicly sharing data—e.g. participant privacy or use of data from a third party—those must be specified.

YES

*4. Is the manuscript presented in an intelligible fashion and written in standard English?

PLOS ONE does not copyedit accepted manuscripts, so the language in submitted articles must be clear, correct, and unambiguous. Any typographical or grammatical errors should be corrected at revision, so please note any specific errors here.

YES

***5. Review Comments to the Author**

Please use the space provided to explain your answers to the questions above. You may also include additional comments for the author, including concerns about dual publication, research ethics, or publication ethics. (Please upload your review as an attachment if it exceeds 20,000 characters) (Limit 200 to 20000 Characters)

Review for Facing Flood Disaster: Assessing Communities' Knowledge, Skills and Preparedness utilizing a Health Model Intervention. (Manuscript Draft)

The review claims that a Health Belief Model-based intervention that incorporates the behavior change theory to influence behaviour changes, and address associated factors, would improve Malaysia's Selangor communities' knowledge, skills, and preparedness for flood disasters.

This is a valid claim noting that The Health Belief Model is a widely applied theory for community-based intervention studies. It has also been found to improve flood disaster preparedness in various countries. The authors mention other possible theories but opt for 'The Health Belief Model' in disaster preparedness efforts. This decision is based on the existing gap in Malaysia's current educational intervention, the model's suitability in focusing on human behaviour and its consequent ability to manage hazard aftermaths.

All data collected and the subsequent analyses support the study claims. The cluster randomized controlled trial method was used to conduct the research.

The paper has followed the CONSORT guidelines but with the following shortcomings:

- The manuscript is not explicitly identified as a cluster randomized trial in the title.
- The source of funding is not given in the abstract.
- 3 of 9 eligible clusters refused to participate in the research due to the coronavirus disease (COVID-19). Exclusion of the 3 clusters, though captured in Figure 1, (a CONSORT flow diagram), is not explicitly recorded in the descriptive text. The authors state a 100% retention of the dataset at baseline, however, that 'original' dataset excludes an eligible 33.3%.

Considering the operative analytical sample, the methodology is technically sound and therefore able to allow reproduction of the experiments. This is based on the following:

- Participants were randomly assigned to a group.
- Both participants and trainee personnel were blinded.
- Questionnaires used were validated.
- There was low risk of contamination between the groups.
- There was no ongoing district program during the time of the research.
- There was 100% retention of the identified analytical sample following exclusion of the 3 above-mentioned clusters.

The experiments were conducted rigorously, and purpose outlined. The necessary adjustments for covariates were done and statistical analyses were performed and presented in detail. The data collected and analyzed support the conclusions that have been drawn.

The authors stated that the data underlying the findings is available without restriction, however, they also state that datasets used and analyzed during the study are available from the corresponding author on **reasonable request**. It is not clear whether that is a restriction to

this review, given that there is no indication of whether the URLs/accession numbers/DOIs will be available only after acceptance of the manuscript for publication.

The manuscript is presented in an intelligible fashion and written in standard English. It can be accessible to non-specialists. However, the following considerations would ensure better organization and clarity across the manuscript:

- Page 5 - line 108 to 109: “Because Malaysians are rarely exposed to major disasters, they are accustomed to them” – The intended connotation of this sentence is unclear.
- Page 7-8: There is repetition between lines 163 and 174
- Page 12 - line 228: The final sample size given as 84. This does not tally with the 284 number referred to under ‘Results and Response Rates’ on line 282 for example. Further clarification is needed.
- Page 13 - line 249 to 256: The paragraph on maturation seems misplaced or not adequately explained.
- Page 10 - Table 1, under methods: “Perceived benefit and perceived benefit were assessed….” “Perceived benefit” is repeated. In addition, the first columns of Tables 7, 8 and 9 require some formatting.
- Figures 1 to 12 require complete labelling. Though they can still be correctly interpreted as presented, explicit labeling of both axis’ is suggested.
- Page 29 - Line 496 and 497: “This HEBI's efficacy was comparable to that of the systematic review in **chapter 2** regarding the optimal dose for ….”. “Chapter 2” is not referenced, and it is unclear where to locate it in the manuscript.
- Line 552 and 553: “The previous systematic review identified interventional studies that use the HBM as the theoretical basis for intervention design.” It is not clear which ‘previous systematic review’ the authors are referring to.
- Page 37 - Line 554 to 556: “However, 14 (78%) of the 18 studies examined showed significant changes in adherence, while 7 (39%) showed small overall result.” There seems to be a possible error. If 14 out of 18 showed significant changes then perhaps it should read 4 (22%). Further clarification may be necessary.

In my opinion, this manuscript does not contain Dual Use Research of concern. Consequent to my search, the study also appears to present results of an original research.

A study protocol by the manuscript authors is available using the following link - https://doi.org/10.1186/s12889-021-11719-3. It is titled as stated below:

A cluster-randomized trial study on effectiveness of health education-based intervention (HEBI) in improving flood disaster preparedness among community in Selangor, Malaysia: a study protocol

The following deviations from the protocol are noted:

- The manuscript makes no mention of data collection at 3 months post-intervention as is stated in the protocol's abstract.
- The protocol indicates that the research will include 6 districts. There was no explicit statement regarding the three that refused to participate due to COVID-19 as stated in the manuscript's flow chart.

Should the paper be considered unsuitable for publication in its present form, the authors should be encouraged to submit a revised version. I will make myself available to answer questions from the editors and for a re-review of the manuscript.

Thank you.
