# Supplementary material for: Facing flood disaster: A cluster randomized trial assessing communities’ knowledge, skills and preparedness utilizing a health model intervention
Source: PLoS One. 2022 Nov 28;17(11):e0271258. doi: 10.1371/journal.pone.0271258 (PMC9704659; doi:10.1371/journal.pone.0271258)
Supplement: Attachment — Submitted filename: Response to Reviewer.docx [file pone.0271258.s005.docx]

We Thank Editor and Reviewers. Below are our responses.

1. ORCID ID: We have added Corresponding Author ORCID ID in the revision system.

2. Figure 2-4 has been depicted in the text.

3. Anonymized Data set has been provided in the revision system.
